# Bioprospecting of Cheese Whey: Isolation, Safety and Cytoprotective Effects of *Limosilactobacillus fermentum* Strains on Intestinal Cells

**DOI:** 10.3390/ijms27198860

**Published:** 2026-10-04

**Authors:** Ladislao Iván Díaz Vergara, Marina del Rosario Bettiol, Antonella María Centomo, Lilia René Cavaglieri, Mariana Angélica Montenegro, Yanina Estefanía Rossi

**Affiliations:** 1 Instituto Multidisciplinario de Investigación y Transferencia Agroalimentaria y Biotecnológica (IMITAB), Consejo Nacional de Investigaciones Científicas y Técnicas (CONICET), Instituto Académico Pedagógico de Ciencias Básicas y Aplicadas, Universidad Nacional de Villa María (UNVM), Villa María 5900, Argentina; ladiazvergara@unvm.edu.ar (L.I.D.V.); marinabettiol@unvm.edu.ar (M.d.R.B.); antocentomo@unvm.edu.ar (A.M.C.); 2 Department of Microbiology and Immunology, National University of Río Cuarto, Córdoba 5800, Argentina; lcavaglieri@exa.unrc.edu.ar

**Keywords:** antioxidant activity, Caco-2, cheese whey, co-aggregation, IEC-18, *Limosilactobacillus fermentum*, oxidative stress, potential probiotics

## Abstract

Bioprospecting is the identification and exploitation of natural resources for commercial purposes. Cheese whey is a major dairy by-product rich in nutrients and a possible source of bioactive compounds. The aim of this study was to isolate and identify lactic acid bacteria from cheese whey and characterize them based on their probiotic and technological properties, together with their cytotoxicity and cytoprotective effect on intestinal epithelial cells. From 142 isolates, three were selected as potential probiotic strains; all three were identified as *Limosilactobacillus fermentum* by 16S rRNA gene sequencing. The strains co-aggregated with pathogenic bacteria (up to 52.7%), scavenged the ABTS^●+^ radical cation (10–70%), produced 374–459 mg/L of exopolysaccharides and tolerated sucrose, NaCl and CaCl_2_ at the concentrations tested. *L. fermentum* VM016, VM023 and VM030 showed no cytotoxicity at 10^7^ CFU/mL and showed a cytoprotective effect against the oxidative stress of menadione at concentrations as low as 10^4^ CFU/mL in IEC-18 cells, and 10^5^ CFU/mL in Caco-2 cells. These results show that cheese whey retains a cultivable lactic acid bacteria fraction already adapted to the physicochemical conditions of the dairy matrix, from which autochthonous cultures can be recovered exhibiting probiotic potential, technological suitability, and favorable preliminary safety indicators for human consumption.

## 1. Introduction

In recent years, consumers have increasingly sought novel foods with health-promoting properties and from natural sources [1]. This has led the food industry to search for new ingredients with which to enrich their products to satisfy consumer demand. Bioprospecting, defined as the identification and exploitation of natural resources for commercial purposes, has become a valuable tool for developing new ingredients obtained from natural sources [2].

Cheese whey is a dairy by-product obtained from cheese manufacture. It has a high nutritional value, mainly attributed to proteins and to their reported health benefits such as enhanced immunity, antioxidant activity, anticancer effects against colon cancer, etc. [3,4].

Probiotics are live microorganisms that, when properly administered, confer a health benefit on the host [5]. Although this is not a universal criterion, a minimum viable count of 10^6^ colony-forming units per milliliter (CFU/mL) or per gram of food at the time of consumption is frequently suggested as a practical reference for probiotic foods [6]. To exert their beneficial effect, probiotics must be resistant to low pH and bile to survive in the upper digestive tract and subsequently colonize by adhering to intestinal epithelial cells. Because safety-related traits are species- and often strain-dependent, they must be assessed for each candidate strain rather than assumed for LAB as a group; in this context, an important characteristic to consider when characterizing potential probiotic strains is their susceptibility to antibiotics [7].

Several lactic acid bacteria (LAB) strains have demonstrated probiotic properties, but only some have been declared as such [8]. Among the LAB, *Limosilactobacillus* (formerly *Lactobacillus*) is a relatively new genus composed of 31 species, most of which were isolated from intestinal or fecal samples; some species, such as *L. reuteri* and *L. fermentum*, are produced commercially as starters and probiotics [9]. *L. fermentum* can be found in various habitats, such as vegetables, cereal-based fermented foods, the oral cavity, human breast milk and in the vagina. This microorganism has been extensively studied over the years for various applications and has demonstrated numerous beneficial properties in various in vitro and in vivo models [10]. *L. fermentum* stands out for its ability to promote the growth of beneficial intestinal microorganisms and to improve immunity, among other properties [11]. The isolation of *L.* fermentum from whey has been documented in a few studies [12,13,14,15,16]. Some *L. fermentum* isolates have shown promising traits such as acid and bile salt resistance, antimicrobial activity and bacteriocin production [12,15]. Nevertheless, to our knowledge, no study has yet reported an integrated characterization covering probiotic potential, preliminary safety and technological aptitude of *L. fermentum* isolates recovered from cheese whey, nor has their cytoprotective effect on intestinal epithelial cells under oxidative stress been evaluated. The present work addresses this gap.

Probiotic properties must be confirmed through in vivo trials to demonstrate their beneficial effects. However, before these experiments are conducted, in vitro evaluations of their safety and bioactivity must be carried out. Therefore, the hypothesis of this work is that cheese whey harbors autochthonous strains with probiotic potential, favorable preliminary safety indicators, technological aptitude for food applications, and cytoprotective effects on intestinal cells exposed to oxidative stress. To test this hypothesis, the present study aims to conduct bioprospecting of cheese whey by isolating and characterizing microorganisms with probiotic and technological properties, as well as evaluating their cytotoxicity and cytoprotective effects on different cell lines under stress conditions.

## 2. Results

### 2.1. Isolation, Characterization and Identification of Potential Probiotic Microorganisms

A total of six whey samples were obtained. After an enrichment in MRS broth, the samples were seeded in MRS agar, and 142 bacterial colonies were isolated. After Gram staining and catalase test, all Gram-negative and catalase-positive isolates were discarded, leaving 64 isolates for further analysis. These 64 isolates were assessed spectrophotometrically for their auto-aggregation capacity. Based on the previous studies, an auto-aggregation value of 29% was taken as the cut-off point, and 44 isolates were discarded.

Hemolytic activity in bacteria results from the lysis of red blood cells and is considered a virulence trait. None of the 20 remaining isolates exhibited hemolytic activity.

**Figure 1:**
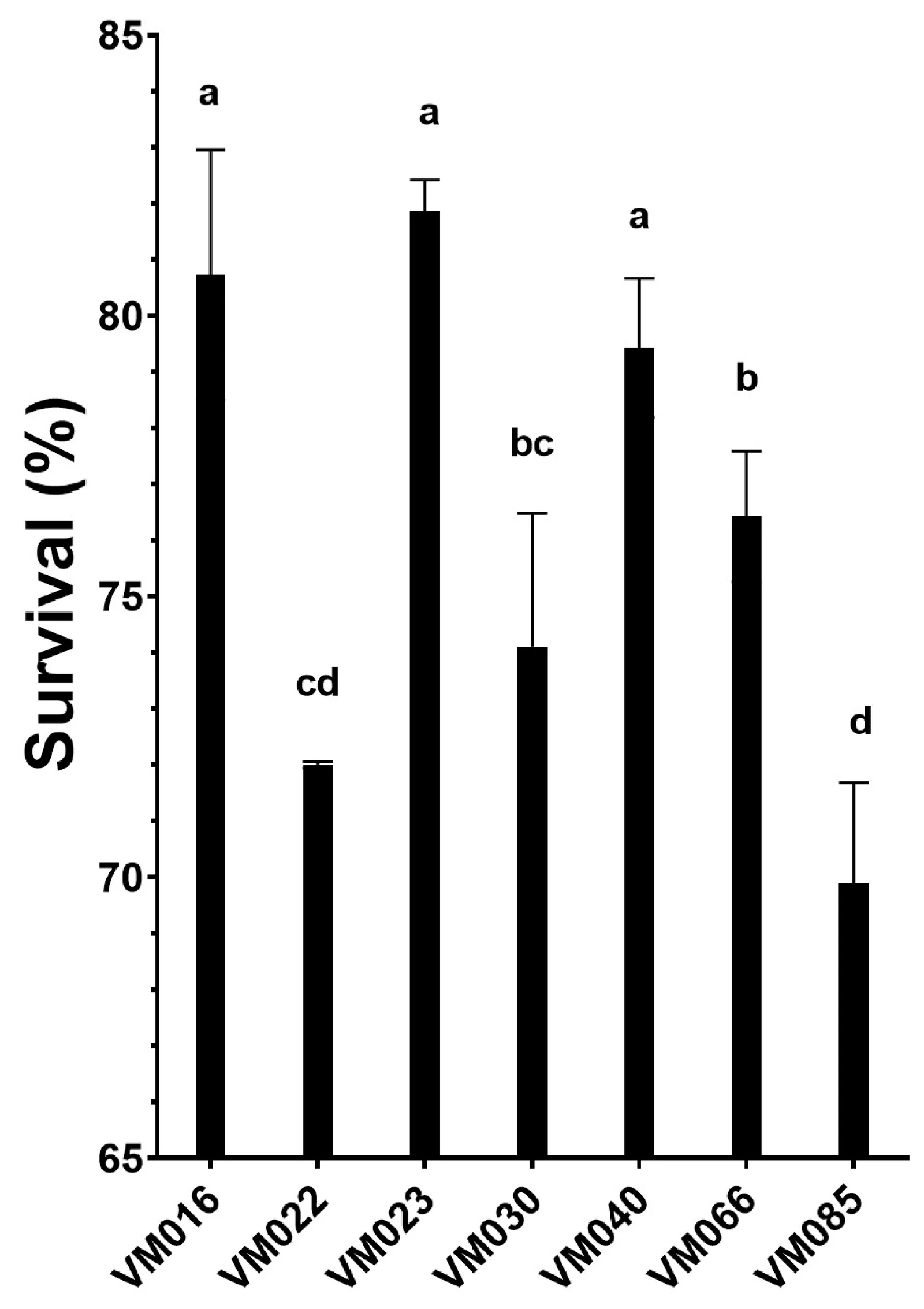
Survival (%) of the seven isolates that tolerated sequential exposure to acid (pH 2, 2 h) and bile salt (0.45% *w*/*v* pH 8, 4 h) conditions relative to the initial inoculum. Values are presented as mean ± SD (n = 3). Bars sharing the same letter are not significantly different according to Fisher’s LSD test (*p* < 0.05).

When selecting potential probiotic microorganism, tolerance to acid and bile salts constitutes one of the most decisive criteria. Only 7 of the 20 evaluated isolates (VM016, VM022, VM023, VM030, VM040, VM066 and VM085) survived the sequential acid and bile salt challenges. The isolates that survived these conditions showed a reduction of 1 to 2 Log CFU/mL, indicating good tolerance to acid and bile stress (Figure 1). Nevertheless, isolate VM085 was discarded because its lower survival rate was below the 70% threshold.

The preliminary screening for antibiotic susceptibility was used as the final selection criterion for the PPMs and was interpreted from the inhibition zone diameters according to the manufacturer’s interpretive criteria (Section 4.3.4).

Of the six isolates evaluated, three (VM022, VM040 and VM066) were resistant to all 10 antibiotics tested and were discarded for the subsequent trials. Isolates VM016, VM023 and VM030 showed the lowest resistances (Table 1). These bacteria were selected for molecular identification and characterization of their probiotic properties.

Based on the analysis of the partial sequence of 16S rRNA gene (approx. 1400 bp), the three isolates (VM016, VM023 and VM030) were identified as *Limosilactobacillus fermentum* (basionym: Lactobacillus fermentum Beijerinck 1901); in all three cases the homology coverage was 100% across the full length of the gene fragment studied, corroborating the identity of the three sequenced fragments using the AlignX global alignment tool from the Vector NTI Advance 10.3.0 package. 16S rRNA sequences were deposited in the Gene Bank under the accession numbers PZ954042, PZ954049 and PZ45047 for *L. fermentum* VM016, *L. fermentum* VM023 and *L. fermentum* VM030, respectively.

### 2.2. Probiotic Properties

The antimicrobial activity of the three *L. fermentum* strains evaluated against nine pathogenic bacteria is shown in Table 2. *L. fermentum* VM016 inhibited six of the nine indicator strains, *L. fermentum* VM023 four and VM030 three. None of the strains inhibited *Salmonella* LM001 or *Bacillus cereus* ATCC 11771.

The co-aggregation assay is another antagonistic activity against pathogenic bacteria. The three selected *L. fermentum* strains co-aggregated with some of the pathogenic bacteria tested. The co-aggregation values obtained ranged from 0 to 52.7% (Table 3).

The antifungal activity is shown in Table 4. *L. fermentum* VM016 completely inhibited *Aspergillus niger* and *Rhizopus* sp., and *L. fermentum* VM023 partially inhibited both, whereas VM030 inhibited neither. In the case of *Penicillium roqueforti*, none of the strains studied inhibited its growth. This may represent a technological advantage, since this fungus is used in the dairy industry for cheese production.

**Figure 2:**
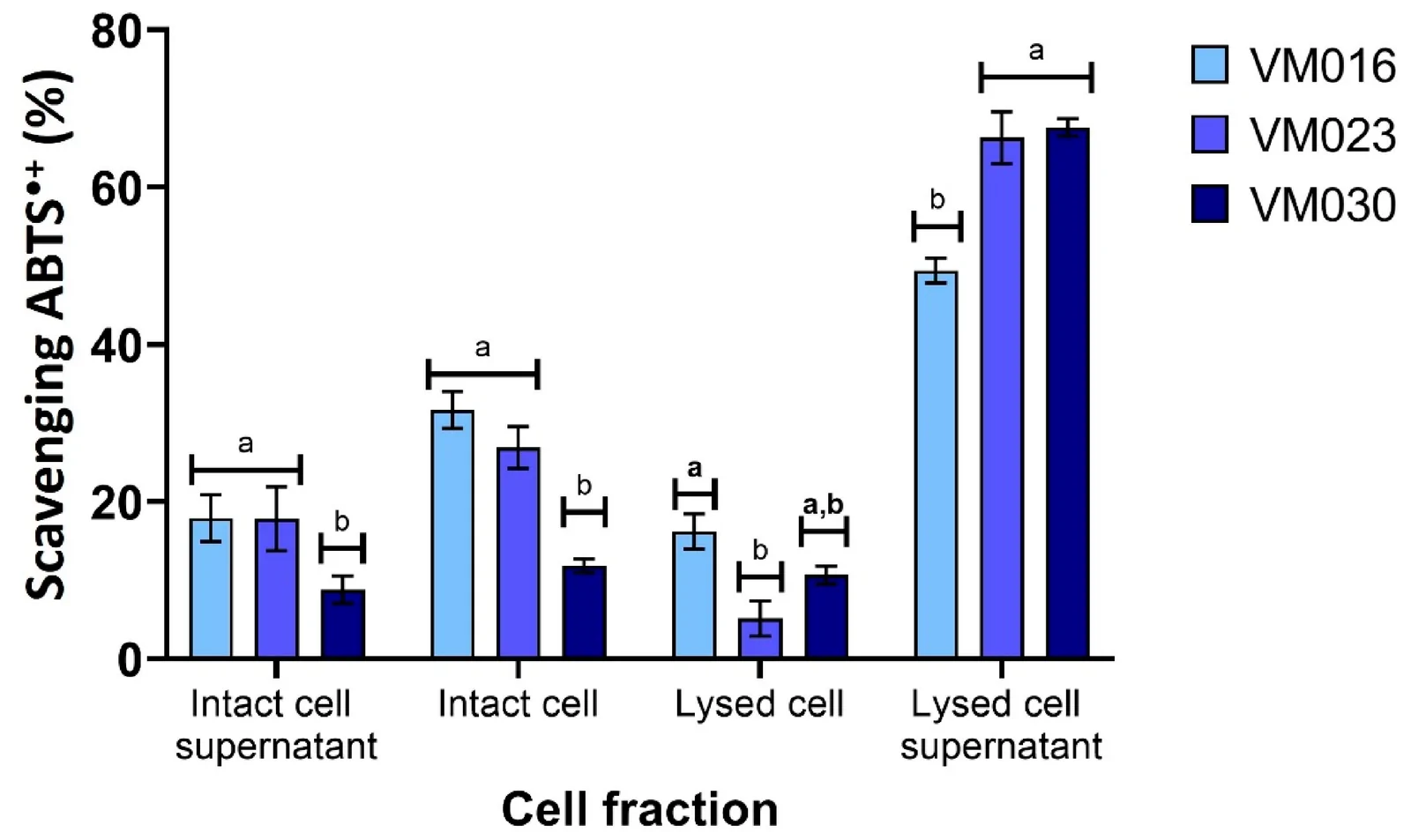
ABTS^●+^ radical cation scavenging (%) of intact and lysed cells of *Limosilactobacillus fermentum* VM016, VM023 and VM030, and of their respective supernatants. Values are presented as mean ± SD (n = 3). Within each cell fraction, strains were compared by one-way ANOVA; bars sharing the same letter are not significantly different according to Fisher’s LSD test (*p* < 0.05).

The ABTS^●+^ radical cation scavenging assay was used to evaluate the antioxidant capacity of the *L. fermentum* strains studied. All cell fractions exhibited ABTS^●+^ scavenging activity (Figure 2). For intact cells, VM016 and VM023 did not differ significantly from one another, but both were significantly more active than VM030, which showed the lowest activity. The lysed-cell supernatant was the most active fraction for every strain: VM023 and VM030 reached the highest values, close to 70%, with no significant difference between them, whereas VM016 had significantly lower values, at approximately 50%.

Exopolysaccharide production was 374.07 ± 57.62, 459.26 ± 41.90 and 433.33 ± 15.71 mg/L for *L. fermentum* VM016, VM023 and VM030, respectively.

**Figure 3:**
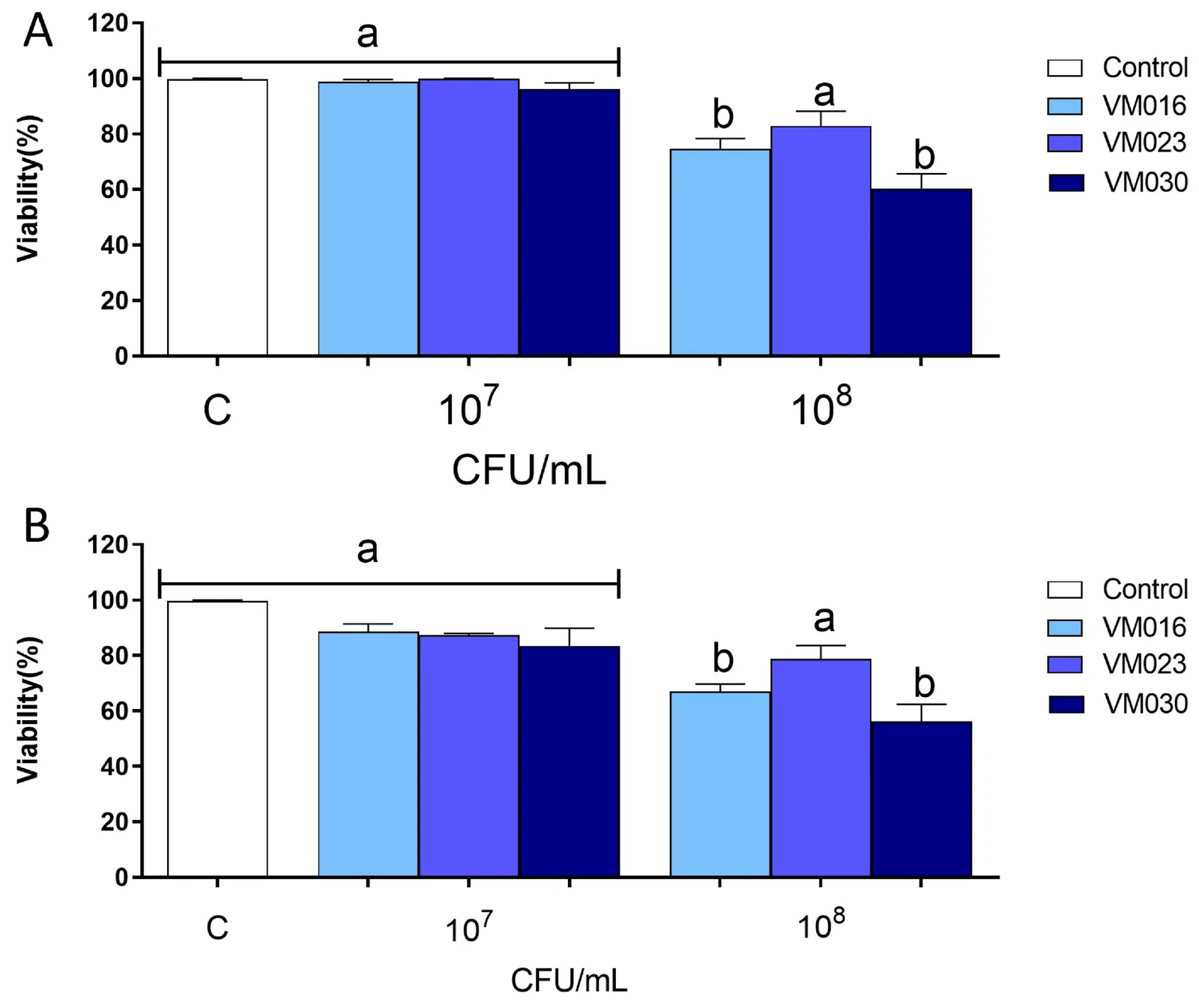
Cell viability of IEC-18 (**A**) and Caco-2 (**B**) treated with the three *Limosilactobacillus fermentum* strains at 10^7^ and 10^8^ CFU/mL. Control cells were incubated in unsupplemented DMEM. Values are expressed as percentage of the control (mean ± SD of three independent experiments, n = 3). Within each cell line, all strain–concentration combinations and the control were compared together by one-way ANOVA followed by Bonferroni’s post hoc test; bars sharing the same letter are not significantly different (*p* < 0.05).

The effect of the three strains of *L. fermentum* VM016, VM023 and VM030 on the viability of IEC-18 and Caco-2 cells is shown in Figure 3A and 3B, respectively. At 10^7^ CFU/mL none of the strains reduced viability relative to the untreated control in either cell line, indicating the absence of cytotoxic activity. At 10^8^ CFU/mL, viability of both cell lines was significantly reduced after exposure to VM016 and VM030 (to approximately 60–80% of the control), whereas VM023 maintained viability comparable to the control.

**Figure 4:**
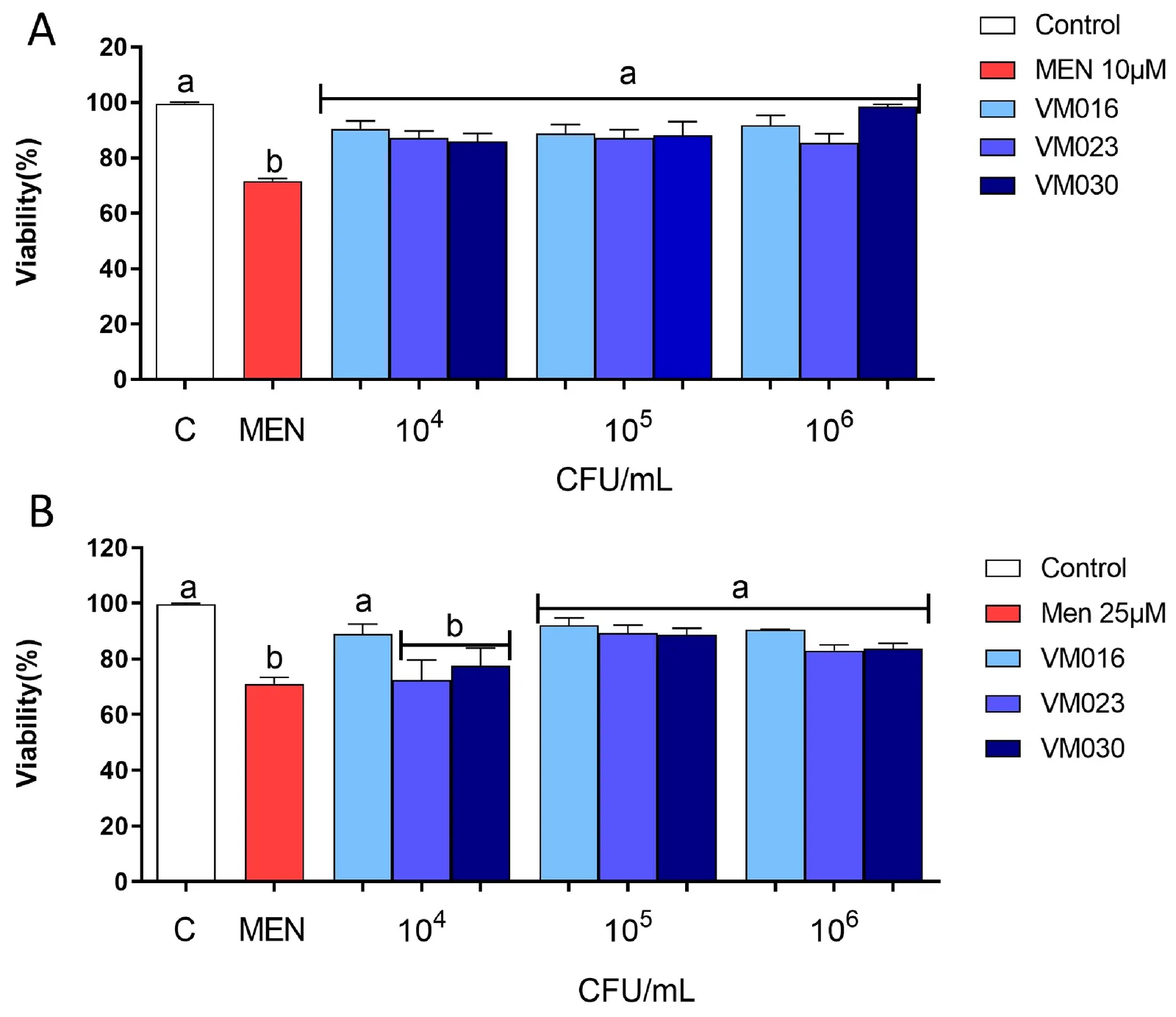
Cytoprotective effect against MEN-induced oxidative stress after co-treatment with *Limosilactobacillus fermentum* VM016, VM023 and VM030. (**A**) IEC-18 cells co-treated with MEN (10 µM) and bacteria at 10^4^–10^6^ CFU/mL. (**B**) Caco-2 cells co-treated with MEN (25 µM) and bacteria at 10^4^–10^6^ CFU/mL. Control cells were incubated in unsupplemented DMEM. Values are expressed as percentage of the control (mean ± SD of three independent experiments, n = 3). Within each cell line, all treatments were compared together by one-way ANOVA followed by Bonferroni’s post hoc test; bars sharing the same letter are not significantly different (*p* < 0.05).

The cytoprotective effect of *L. fermentum* VM016, VM023 and VM030 against oxidative stress generated by MEN in both cell lines was evaluated (Figure 4). Co-treatment with *L. fermentum* VM016 or VM023 preserved the viability of IEC-18 cells above 81% across the whole range assayed, whereas VM030 was protective only at concentrations of 10^6^ CFU/mL or below (Figure 4A). In contrast, *L. fermentum* VM030 was cytoprotective only at concentrations equal to or lower than 10^6^ CFU/mL (Figure 4A).

In Caco-2 cells, co-treatment with 25µM MEN and *L. fermentum* VM023 or VM030 was cytoprotective at concentrations between 10^5^ and 10^6^ CFU/mL with viabilities above 82%. While *L. fermentum* VM016 protected cells at a lower concentration, 10^4^ CFU/mL (Figure 4B) had a greater cytoprotective effect.

### 2.3. Technological Properties

The technological properties of the evaluated strains are summarized in Table 5. None of the three strains showed extracellular proteolytic or lipolytic activity. Regarding sugar tolerance, *L. fermentum* VM016 was the only strain whose growth was not affected (growth index GI > 75%) at any of the sucrose concentrations evaluated up to 25% *w*/*v*; growth of VM023 and VM030 was moderately reduced (25% < GI < 75%) at all sucrose concentrations tested, although neither was strongly inhibited. With respect to NaCl tolerance, growth of the three strains was unaffected at 2 and 4% *w*/*v*. Likewise, the presence of CaCl_2_ in the medium did not affect the growth of the *L. fermentum* strains.

All strains grew at the three pH values evaluated (GI > 75% for VM016 and VM023; 25% < GI < 75% for VM030). The *L. fermentum* strains have good tolerance to low pH, and their growth was most favored at pH close to 6, the typical pH of the whey from which they were isolated. The three strains were able to grow at 37 °C and 20 °C, but not at 8 °C or 50 °C, and none was inhibited by the vanilla and strawberry essences at 0.1% *v*/*v*.

## 3. Discussion

A total of 142 bacterial colonies were obtained, and a stepwise selection process was applied to identify the best candidates. Since the aim of this study was to isolate LAB with probiotic properties, only the 64 Gram-positive, catalase-negative isolates were tested for auto-aggregation. This analysis plays an important role when studying probiotics since auto-aggregation is generally related to cell surface hydrophobicity and adhesion properties, which is also associated with its ability to survive and persist in the gastrointestinal tract [17]. Aziz et al. studied the probiotic characteristics of Lactobacillus species isolated from chicken intestine, indicating that an auto-aggregation between 20 and 50% can be considered moderate [18]. Furthermore, Rodríguez-Sánchez et al. considered values at or below 10% to indicate an absence of aggregation [19]. With the cut-off point of 29%, 20 strains with greater auto-aggregation were selected to test their hemolytic activity. Bacteria should not exhibit hemolytic activity to be considered probiotics [20]. Although it is uncommon, Zhou et al. indicated that some LAB exhibit hemolytic activity, producing erythrocyte lysis and promoting the penetration of virulent pathogens and cytotoxicity [21]. Therefore, when considering safety, the absence of hemolytic activity is important in the selection of potential probiotic strains. None of the evaluated strains showed hemolytic activity.

One of the most important characteristics of probiotic strains is their capability to survive in gastrointestinal conditions. Marquez et al. studied the probiotic potential of eight *Lactobacillus* strains from goat dairy products and reported survival between 59.7 and 85.1% [22]; the survival of 70 to 82% observed here for the seven retained isolates (Figure 1) falls within that range, indicating comparable tolerance. Terpou et al. indicate that the adaptation of LAB to acid stress is related to proton translocation mechanisms, changes in membrane fatty acid composition, cytoplasmic alkalinization due to the action of various enzymes, and the production of stress proteins, among other physiological changes and structural components of the cells [23]. The VM085 strain had the lowest survival rate (Figure 1) among the seven isolates and was therefore discarded from further analysis.

In recent years, antibiotic-resistant bacteria have become an important health concern, reducing the efficacy of medications [24]. It should be noted that disk diffusion on MRS agar is not a standardized method for lactobacilli and that the interpretive criteria applied (manufacturer-derived, based on clinical breakpoints) cannot discriminate intrinsic from acquired resistance. The profile reported in Table 1 should therefore be regarded as a preliminary screening that still provides useful information for initial selection. Antibiotic resistance is a natural survival strategy that can be expressed in all microorganisms. In probiotic microorganisms the presence of transferable antibiotic resistance is a negative characteristic, because they may transfer this antibiotic resistance to potentially pathogenic bacteria [25]. Islam et al. indicated that antibiotic resistance is one of the most important parameters to consider when assessing the safety of a microorganism [26]. On the other hand, Vieira de Souza et al. indicated that, although some strains may show resistance to vancomycin, this is generally not a concern when the resistance is intrinsic and chromosomally encoded, as described for several LAB species [27]. Therefore, this resistance will not be transferable to pathogens. Intrinsic vancomycin resistance has been widely reported in heterofermentative lactobacilli, including *L. fermentum*, and is generally considered non-transferable; however, because the genetic basis of the vancomycin resistance observed in VM030 was not investigated in this study, this interpretation should be considered tentative until confirmed at the strain level. Among the six evaluated strains, VM016, VM023 and VM030 were the strains that presented the lower antibiotic resistance and were selected for molecular identification.

The three strains were identified as *L. fermentum*, a species commonly isolated from dairy products, such as cheese curd and raw milk, but seldom reported in whey. Rzepkowska et al. isolated 25 LAB strains from organic cheese whey, of which nine were identified as *L. fermentum* and seven as *L. plantarum* [28]; this remains one of the few precedents for the present findings.

An important health claim associated with probiotics is the prevention of infectious disease in the gastrointestinal tract. The antagonistic activity of *L. fermentum* can inhibit the growth or colonization of pathogenic bacteria [29]. LAB produces a range of antimicrobial agents, such as organic acids, hydrogen peroxide, and bacteriocin-like peptides, which could explain the antimicrobial activity observed against the pathogenic bacteria evaluated. Co-aggregation with pathogens is also considered an important characteristic for potential probiotic strains because it can help prevent the colonization of pathogenic bacteria in the gastrointestinal tract [19]. Rodríguez-Sánchez et al. reported co-aggregation between 0 and 55.3% for *Lactobacillus* strains with antimicrobial activity confronted with *Salmonella choleraesuis* CECT 443 and *Staphylococcus aureus* CECT 86 [19], a range comparable to that obtained here, although co-aggregation values are strongly dependent on the method and the pathogen tested, and such comparisons should be treated with caution. Mkadem et al. indicated that the co-aggregation activity of LAB, and of probiotics in particular, is considered an important attribute for competitively inhibiting the adherence of pathogenic bacteria to the gastrointestinal tract [30].

The antifungal activity observed in *L. fermentum* strains may be associated with the production of lactic acid, acetic acid or phenyl lactic acid [31]. The absence of activity against *P. roqueforti* is of practical interest, since this species is used in the manufacture of mold-ripened cheeses and compatibility with it would be a prerequisite for such applications. The EPS production is relevant both technologically, through its effect on texture and water retention, and biologically, through reported prebiotic, immunomodulatory and antioxidant properties [32,33].

Another key characteristic of probiotic microorganisms is the preservation of intestinal health through the protection of the intestinal mucosal barrier integrity or enterocyte health. Oxidative stress can damage cells and tissues. Although humans possess an endogenous antioxidant defense system, exogenous antioxidants are sometimes required to counteract oxidative stress [34]. The antioxidant activity of *L. fermentum* strains could therefore contribute to intestinal cell health. *L. fermentum* intact-cell antioxidant activity is attributed to surface compounds in the bacterial cell wall, where a possible interaction with the cell interior may regulate the access of the ABTS^●+^ radical cation. Among the intact cells, *L. fermentum* VM016 showed the highest value. Cell lysis of the strains increases the antioxidant activity by allowing the interaction between the cation ABTS^●+^ and the internal cell compounds, which is consistent with Liu et al. [35], who studied cell-free extracts of *Lactobacillus* and found that suspensions retaining a larger proportion of intact cells after disruption displayed higher antioxidant activity. When the *L. fermentum* cells were lysed and centrifuged, however, the water-soluble fraction (peptides, amino acids, sugars) was separated from the insoluble one (cell wall, membrane), and it is in this lysed-cell supernatant that antioxidant activity was highest relative to the intact-cell supernatant. This is consistent with Vitheejongjaroen et al. [36], who examined viable cells, cell-free supernatant and cell-free extract of *Bifidobacterium* and proposed that probiotics may contain intracellular bioactive compounds which, once released by lysis, react effectively with free radicals and convert them into more stable products, terminating the radical chain reaction. Taken together, these observations suggest that the compounds responsible are largely water-soluble and located intracellularly [37,38].

The cytotoxicity of the *L. fermentum* strains VM016, VM023 and VM030 was evaluated in the IEC-18 and Caco-2 cell lines. In these cell lines the cytotoxicity at 10^8^ CFU/mL observed for VM016 and VM030 can be classified as moderate, because cell viability is between 60 and 80% [39]. It should be stressed, however, that a moderate reduction in cell viability at this concentration does not support a general safety conclusion, and that results from in vitro cell viability assays cannot be directly translated into safety for human consumption; these data are presented only as a preliminary safety indicator. It is also worth noting that VM023 did not reduce viability even at 10^8^ CFU/mL. In contrast, no cytotoxic effect was detected at 10^7^ CFU/mL for any strain, and the concentrations used in the cytoprotection assays (10^4^–10^6^ CFU/mL) were below this level. Therefore, the three *L. fermentum* strains can be considered non-cytotoxic at the concentrations used to evaluate their cytoprotective effect. MEN exert a cytotoxic effect on cells through its interaction with thiol groups on essential molecules and the redox cycling that results in H_2_O_2_ [40,41]. The cytoprotective effect observed for *L. fermentum* strains may be related to the antioxidant activity determined in this work. It is plausible that the antioxidant activity of both intact cells and metabolites contributes to reducing menadione-induced oxidative stress, thereby protecting IEC-18 and Caco-2 cells from cytotoxicity. However, as the underlying mechanism was not directly investigated, the potential intestinal health benefit of these strains should be regarded as a possible explanation rather than a demonstrated mechanism, and any intestinal health benefit remains to be confirmed in vivo using appropriate animal models.

Since no predefined weighting of the selection criteria was applied, the three strains were compared qualitatively across all assays. Among the three *L. fermentum* strains, VM016 stands out as the most promising candidate due to its broader antimicrobial and antifungal activity, inhibiting six of the nine indicator bacteria and completely inhibiting two of the three fungi tested. Furthermore, *L. fermentum* VM016 showed, together with VM023, the highest ABTS^●+^ scavenging activity of intact cells, and demonstrated the most efficient cytoprotective effect against oxidative stress in Caco-2 cells, preserving cell viability even at the lowest concentration tested (10^4^ CFU/mL). Additionally, VM016 was the only strain whose growth was unaffected at the highest sucrose concentration tested. It should be noted, however, that VM023 and VM030 outperformed VM016 in some assays (e.g., antioxidant activity of the lysed-cell supernatant, co-aggregation with Salmonella LM001 and, for VM023, absence of cytotoxicity at 10^8^ CFU/mL); the ranking proposed here is therefore qualitative and would benefit from an integrated scoring approach in future work.

Limitations of the study. Several limitations of this work should be acknowledged. First, the probiotic and safety characterization relied exclusively on in vitro assays; the strains are therefore presented as strains with probiotic potential, and in vivo studies are required before any health claim can be made. Second, species identification was based on 16S rRNA gene sequencing, which has limited resolution within the Lactobacillaceae; sequencing of housekeeping genes (e.g., *pheS*, *rpoA*; Naser et al. [42]) or whole-genome sequencing would provide a more robust identification and a genome-based safety assessment, including the detection of acquired antibiotic resistance and virulence genes. Third, antibiotic susceptibility was assessed only by a preliminary disk diffusion screening, and MIC values interpreted with microbiological cut-off values remain to be determined. The antibiotic panel used did not fully coincide with the antimicrobials recommended by EFSA guidance for Lactobacillaceae [43]. Fourth, the acid and bile assays were simplified challenges that did not include digestive enzymes and thus do not fully reproduce gastrointestinal transit. Fifth, although the samples covered three production lines and were collected on different occasions, all of them came from a single dairy facility, which limits the generalization of the findings to other cheese-making environments. Finally, no actual food matrix was tested, and survival of the strains during processing and storage remains to be evaluated.

## 4. Materials and Methods

### 4.1. Sample Collection

Whey samples were obtained from Cooperativa Agrícola Ganadera de Arroyo Cabral (Arroyo Cabral, Córdoba, Argentina). Samples were collected from the three production lines of the facility (soft, semi-hard and hard cheeses) at two points of each line: the cheese-making vat and the pasteurizer feed tank. The six samples were collected on different production days, and all samples were kept at 4 °C from collection until analysis.

### 4.2. Isolation of Potential Probiotic Microorganisms

Potential probiotic microorganisms (PPM) were isolated according to Abushelaibi et al. [44]. Initially, whey samples were incubated at a 1:10 *v*/*v* sample-to-medium ratio in De Man, Rogosa and Sharpe (MRS) broth (Britania, Argentina) at 37 °C for 48 h under microaerophilic conditions. Bacterial isolation was performed by streaking on MRS agar, and the plates were incubated under the same conditions mentioned above. Colonies with different macroscopic characteristics, such as shape and color, were purified by re-streaking in fresh MRS agar. The isolates were subjected to Gram staining and the catalase test, and were preserved in 25% *v*/*v* glycerol suspension at −20 °C.

### 4.3. Preselection of Potential Probiotic Microorganisms

#### 4.3.1. Auto-Aggregation Assay

The auto-aggregation assay was determined using the modified protocol of Mohd-Zubri et al. [45]. PPM suspensions were grown for 48 h in an MRS broth and harvested by centrifugation at 12,320× *g* for 10 min. The resulting pellets were resuspended in PBS buffer (pH 7.4), adjusted to an optical density of 0.55–0.60 at 600 nm and the aggregation capacity was evaluated after 2 h of incubation at 37 °C. Auto-aggregation values were calculated according to Equation (1).

(1) $$\%\;Autoaggregation=\left[\;1-\frac{A_{t}}{A_{0}}\right]\times 100$$

where *A_t_* is the absorbance after the 2 h of incubation and *A*_0_ represents absorbance at t = 0.

#### 4.3.2. Hemolytic Activity Assay

Hemolytic activity was determined by using blood agar containing 5% (*w*/*v*) sheep blood [20]. The PPMs were seeded and incubated under microaerophilic conditions at 37 °C for 48 h. After incubation, the hemolytic activity of isolated strains was evaluated and classified based on the lysis of red blood cells in the medium around the colonies. No green zones or clear zones around colonies are considered safe.

#### 4.3.3. Tolerance to Acid and Bile Salts

Simplified acid and bile salt challenges were prepared according to Díaz-Vergara et al. with modifications [46]. The previously incubated PPMs in MRS broth were diluted in PBS (pH 7.4) to an initial concentration of 10^7^ CFU/mL. Then, 100 µL of the PPM solution was inoculated into 900 µL of 0.5% *w*/*v* NaCl pH 2, simulating gastric conditions, and incubated for 2 h at 37 °C at 200 rpm under microaerophilic conditions. Once the simulated gastric stage was completed, the PPMs were centrifuged for 5 min at 12,320× *g* and the supernatant was replaced with 0.5% *w*/*v* NaCl containing 0.45% *w*/*v* bile salt at pH 8 to simulate intestinal conditions, and incubated for 4 h under the same conditions. Because neither stage contained digestive enzymes, this model represents a simplified acid and bile challenge rather than a comprehensive simulation of gastrointestinal conditions. Samples were taken before and after each stage and used for microbiology count on MRS agar at 37 °C for 48 h under microaerophilic conditions. Survival (%) was calculated relative to the initial inoculum, and a survival threshold of 70% was adopted as the cut-off criterion for retaining isolates, following previous reports [37].

#### 4.3.4. Antibiotic Susceptibility Test

A preliminary screening for antibiotic susceptibility was determined by the disk diffusion method, using multiple susceptibility disks for Gram-positive microorganisms (Brizuela-Lab, Córdoba, Argentina) [38]. Two multiple disks were used in the trial: series A—vancomycin (30 μg), ciprofloxacin (5 μg), amoxicillin and clavulanic acid (20/10 μg), penicillin (10U) and oxacillin (1 μg); and series B—clindamycin (2 μg), rifampicin (5 μg), gentamicin (10 μg), trimethoprim and sulfamethoxazole (1.25/23.75 μg) and erythromycin (15 μg). The PPM suspension was adjusted to a concentration equal to 10^8^ CFU/mL using the McFarland scale, the strains were seeded on the surface in MRS agar, and the A and B series antibiotic disks were used. After 48 h of incubation at 37 °C, the diameters of the inhibition zones were measured, and the results were expressed as resistant or susceptible according to the zone-diameter interpretive criteria supplied by the manufacturer (Brizuela-Lab). No CLSI or EUCAST breakpoints were applied, since validated disk-diffusion breakpoints are not available for *L. fermentum*; the assay was therefore used only as a preliminary screening (Section 3).

### 4.4. Molecular Identification of Potential Probiotic Microorganisms

The samples were purified, amplified and sequenced by the Genomics Unit of the Institute of Agrobiotechnology and Molecular Biology (IABIMO, CONICET-INTA, Córdoba, Argentina). Following the procedure described by Díaz-Vergara et al., with some modifications, each PPM was inoculated in 10 mL of MRS broth and incubated for 48 h at 37 °C under microaerophilic conditions [46]. Subsequently, it was centrifuged (12,320× *g* for 10 min) and the obtained pellet was resuspended in PBS buffer (pH 7.4) and refrigerated. Bacterial DNA extraction was performed using hexadecyltrimethylammonium bromide (CTAB).

16S rDNA amplification was carried out using the universal primers 16F: 5′-AGAGTTTGATCMTGGCTCAG-3′ and 16R: 5′-TACCTTGTTAGGACTTCACC-3′ [17]. These universal primers allowed the amplification of a fragment of approximately 1450 base pairs (bp).

PCR amplification was performed with 20–30 ng of bacterial DNA in a total volume of 25 μL of 1 × reaction buffer containing 2 mM MgCl_2_, 1.25 U Taq DNA polymerase (5 U/μL, Invitrogen by Life Technologies, Buenos Aires, Argentina), 0.2 mM of each dNTP and 0.6 μM of each primer (16F and 16R). A negative control, containing all reagents without DNA, was included in every set of reactions. PCR was conducted according to the following cyclic conditions: initial denaturation at 94 °C for 3 min, followed by 35 cycles consisting of 94 °C for 45 s, 54 °C for 45 s and 72 °C for 1 min, and a final extension step of 72 °C for 10 min, followed by a hold at 4 °C indefinitely.

Sequence identification was performed using the Vector NTI 10.3.0 program (Invitrogen Corporation 2006) to assemble the complete fragment (contig) from the .ab1 files (electropherograms). The electropherograms were visually inspected to corroborate the base estimate. BLASTn (BLAST+ version 2.13.0; NCBI) (local alignment) software was used for comparison with the GenBank nr database, and the percentage identity, query coverage and closest type-strain sequence were recorded for each isolate.

### 4.5. Characterization of Probiotic Properties

#### 4.5.1. Antimicrobial Activity Assay

The assay was carried out following the description of Abushelaibi et al. with some modifications [44]. PPMs were plated by marking a central streak on MRS agar and incubated at 37 °C for 48 h under microaerophilic conditions. Then, a thin layer of nutrient agar was added and the different pathogenic bacteria (*Escherichia coli* 81382, *E. coli* 81749, *E. coli* ATCC 35218, *Salmonella* spp., *Salmonella* LM006, *Salmonella* LM001, *Staphylococcus aureus* LM005, *S. aureus* ATCC 65389 and *B. cereus* ATCC 11771) were inoculated using a perpendicular streak to the central streak. Antimicrobial activity was determined by the presence of inhibition zones.

#### 4.5.2. Co-Aggregation Assay

PPMs were tested for their capacity to co-aggregate with different pathogenic microorganisms: *E. coli* 81382, *E. coli* 81749, *E. coli* ATCC 35218, *Salmonella* spp., *Salmonella* LM006, *Salmonella* LM001, *S. aureus* LM005, *S. aureus* ATCC 65389 and *B. cereus* ATCC 11771. The co-aggregation assay was conducted according to the protocol established by Prabhurajeshwar and Chandrakanth [47].

The PPMs were cultured in MRS broth for 48 h at 37 °C under microaerophilic conditions. The PPMs were centrifuged at 12,320× *g* for 10 min and the obtained pellet was resuspended in PBS buffer (pH 7.4). Equal volumes of pathogenic and PPM suspensions, with an absorbance between 0.2 and 0.3 at 600 nm, were mixed and incubated for 4 h at 37 °C. To determine the co-aggregation, Equation (2) was used:

(2) % Co−Aggregation = [(Apat + Aprob)/2−Amix]/[(Apat + Aprob)/2]×100

where *Apat* and *Aprob* represent the absorbance at 600 nm of suspensions of the pathogenic and probiotic stain, respectively; *Amix* represents the absorbance at 600 nm of the mixture of suspensions.

#### 4.5.3. Antioxidant Capacity

Antioxidant activity was evaluated by scavenging of the radical cation ABTS^•+^ following Loyeau et al. and expressed as a percentage of scavenging (S%) [48]. Four fractions were assayed: intact cells, intact-cell supernatant, lysed cells and lysed-cell supernatant. Suspensions were adjusted to 1 × 10^8^ CFU/mL and centrifuged (12,320× *g*, 10 min) to separate the cells from the supernatant; intact cells were resuspended in PBS (pH 7.4) before analysis. MRS broth and the lysis buffer were assayed as baselines. For lysis, pellets obtained from 1 × 10^8^ CFU/mL were resuspended in 500 µL of SET buffer (25 mM EDTA, 20 mM Tris, 75 mM NaCl, pH 7) and subjected to three cycles of heating at 100 °C for 5 min and cooling at −4 °C for 5 min. Lysozyme (5 µL, 10 mg/mL) was added and the suspension incubated for 1 h at 37 °C, brought to 1 mL with PBS (pH 7.4) and centrifuged (12,320× *g*, 10 min) to yield the lysed cells and the lysed-cell supernatant.

The ABTS^●+^ solution was adjusted to an absorbance close to 0.9 at 734 nm, and 1 mL of that solution was added to the reaction cuvettes. Subsequently, sample aliquots were added and allowed to react in the dark for 16 min before reading at 734 nm. Percentage of scavenging (*%S*) was calculated as

(3) $$\%\;S=\left[\frac{\left(A_{control}-A_{sample}\right)}{A_{control}}\right]\times 100$$

where *A_control_* is the absorbance of the control and *A_sample_* is the absorbance corresponding, in each case, to the MRS broth in which the bacteria were grown, the lysate solution, intact cells, post-lysate solution and lysed cells.

#### 4.5.4. Antifungal Activity of Potential Probiotic Cultures

The antifungal activity of PPMs was determined using the plate confrontation method described by Zhu et al. [49]. The PPMs were previously incubated in MRS broth for 48 h at 37 °C under microaerophilic conditions. Subsequently, 10 µL of each PPM was inoculated on MRS agar plates following the same incubation conditions mentioned above. The strains were covered with a thin layer of soft malt extract agar (1% agar), 100 µL (10^3^ spore/mL) of the fungal strains was spread over the surface, and the plates were incubated at 28 °C for 72 h. The fungal strains evaluated were *Aspergillus niger*, *Rhizopus* sp. and *Penicillium roqueforti*. Antifungal activity was determined qualitatively, as the presence or absence of inhibition zones.

#### 4.5.5. Exopolysaccharides Production

Exopolysaccharide (EPS) production was determined following the method described by Abid et al. with modifications [50]. The PPMs (0.1 mL of a fresh 48 h MRS culture) were inoculated in ESM culture medium (10 mL) (exopolysaccharide selection medium) (5% *w*/*v* skimmed milk, 0.35% *w*/*v* yeast extract, 0.35% *w*/*v* peptone and 5% *w*/*v* sucrose) and incubated statically (without agitation) at 37 °C for 48 h under microaerophilic conditions. Cultures were centrifuged at 10,956× *g* for 10 min to obtain the supernatant; trichloroacetic acid (TCA) was added at a final concentration of 4% *w*/*v* and the mixture was centrifuged again at 10,956× *g* for 30 min. The clean supernatant was collected, and the EPS was precipitated by adding four volumes of cold absolute ethanol. The solution was kept at 4 °C for 24 h and centrifuged again at 10,956× *g* for 20 min. The precipitate was resuspended in distilled water, and the EPS content was quantified by the phenol–sulfuric acid method at 490 nm against a glucose calibration curve.

#### 4.5.6. Cell Culture and Microorganism Preparation

The IEC-18 cells [51] and Caco-2 [52] cells were obtained from the American Type Culture Collection (ATCC, Manassas, VA, USA) and maintained in Dulbecco’s Modified Eagle Medium (DMEM; Gibco, Grand Island, NY, USA) supplemented with 10% and 20% of fetal bovine serum (FBS; Natocor, Carlos Paz, Argentina,) respectively, 100 U/mL penicillin–100 μg/mL, streptomycin and 1/100 CTS™ GlutaMAX™-I Supplement (Gibco, Grand Island, NY, USA) at 37 °C in a humidified atmosphere of 5% CO_2_ [24].

PPM strains were incubated statically (without agitation) in MRS broth for 48 h at 37 °C under microaerophilic conditions. Cells were then centrifuged at 4 °C, 12,320× *g* for 10 min, the supernatant was discarded, and the pellets were resuspended in PBS. This procedure was repeated twice.

#### 4.5.7. Cytotoxicity Assays

Cellular viability of both cell lines was evaluated by neutral red uptake assay (NR) [53]. IEC-18 and Caco-2 cells were seeded at 5 × 10^4^ cells/well in 96-wells plates and grown for 24 h under maintenance conditions [24]. Then, the cytotoxicity of PPMs was evaluated. Briefly, the medium was discarded and replaced by different concentrations of PPMs diluted in unsupplemented DMEM (10^7^ and 10^8^ CFU/mL) and incubated for 24 h. During co-incubation, medium acidification was monitored through the color of the phenol red indicator of DMEM (which shifts from red to yellow under acidic conditions); no color change was observed in any treatment throughout the incubation period. Bacteria-only blanks (bacterial suspensions in DMEM without cells, processed identically) were included in every plate to exclude interference of bacterial cells with NR uptake and absorbance readings. Then the treatments were discarded and replaced with unsupplemented DMEM containing 90 and 100 µg/mL of NR for IEC-18 and Caco-2 cells, respectively. The plates were incubated for 3 h at 37 °C in the dark, the NR solution was discarded, and the dye was extracted from cells using a mixture of acetic acid, water and ethanol (1:49:50 *v*/*v*/*v*). The absorbance at 570 nm was measured using a microplate spectrophotometer reader Multiskan GO (Thermo Fisher Scientific, Waltham, MA, USA). The cytotoxic effect in both cell types was expressed as the percentage of cell viability with respect to control cells (DMEM). Each condition was assayed in triplicate wells (technical replicates) in each of three independent experiments performed on different days using different cell passages (Section 4.7). Data are shown as the mean ± standard deviation (SD).

#### 4.5.8. Cytoprotection on MEN-Treated IEC-18 and Caco-2

IEC-18 and Caco-2 cells were seeded at 5 × 10^4^ cells/well in 96-wells plates and grown for 24 h under maintenance conditions as previously described, and MEN was used to induce oxidative stress [41]. Cells were treated with MEN at concentrations from 5 to 40 μM for 24 h, and 10 and 25 μM were then selected for IEC-18 and Caco-2 cells respectively (showing 60–70% of cell viability) to examine the cytoprotective effects of PPMs against MEN-induced oxidative stress. Cells were incubated jointly with MEN and PPMs (from 10^4^ to 10^6^ CFU/mL) for 24 h (co-treatment). Cell viability was determined using the NR assay as described previously.

### 4.6. Characterization of Technological Properties

#### 4.6.1. Proteolytic and Lipolytic Activities

To determine the proteolytic activity, plate count agar supplemented with 1% *w*/*v* of skimmed milk powder was used, whereas lipolytic activity was assessed on MRS agar supplemented with 1% *w*/*v* milk cream [54]. The strains were inoculated and incubated for 48 h at 37 °C under microaerophilic conditions. In both cases, the presence of clear zones was used to determine the proteolytic or lipolytic activity.

#### 4.6.2. Tolerance to Sugar, NaCl, CaCl_2_ and pH, and Food Essences

Tolerance to sucrose (10, 15, 20 and 25% *w*/*v*) was evaluated in MRS broth. Tolerance to NaCl was evaluated in MRS agar with 2 or 4% *w*/*v* of NaCl, while the tolerance to CaCl_2_, was evaluated in MRS broth with 1, 2, 4 and 6.5% *w*/*v*. Tolerance to pH was assessed at pH 4, 5 and 6, adjusting the pH of the MRS broth with lactic acid. Tolerance to 0.1% *v*/*v* vanilla or strawberry essence was evaluated in MRS broth. The previously incubated strains were inoculated onto the different media and incubated for 48 h at 37 °C in under microaerophilic conditions, and tolerance was determined following the methodology described by Xu et al. [29] and Aktepe et al. [46] with some modifications. The final absorbance at 600 nm was recorded, and the growth index (*GI*) was calculated according to Equation (4):

(4) $$GI=\left[\frac{A_{sample}}{A_{control}}\right]\;\times 100$$

where A*_sample_* and A*_control_* stands for the absorbance at different sucrose concentration, pH, NaCl concentration or CaCl_2_ concentration values and the control, respectively. Tolerance was classified as (–) GI < 25%: strong inhibition; (+) 25% < GI < 75%: moderate growth; (++) GI > 75%: growth comparable to the control.

#### 4.6.3. Growth at Different Temperatures

The capacity to grow at different temperatures was evaluated in MRS agar [55]. The previously incubated PPMs were inoculated using the streaking technique on MRS agar and incubated for 48 h under microaerophilic conditions at 20, 37, and 50 °C, and for 7 days at 8 °C.

### 4.7. Statistical Analysis

The statistical analysis was conducted using the InfoStat software version 2020 (http://www.infostat.com.ar, accessed on 29 July 2026). Data were analyzed by one-way analysis of variance (ANOVA). For the microbiological assays, means were compared using Fisher’s protected LSD test; in the antioxidant assay, strains were compared separately within each cell fraction. For the cell culture assays, one-way ANOVA was followed by Bonferroni’s post hoc test. A one-way design was chosen because the factor under evaluation was the bacterial treatment: the different strain–concentration combinations were treated as levels of this single factor and were compared together with the untreated control (and with the MEN-only control in the cytoprotection assays), rather than analyzing strain and concentration as two independent factors. Differences were considered significant at *p* < 0.05. Unless otherwise stated, all microbiological assays were performed as three independent experiments (n = 3). Cell culture assays were performed as three independent biological experiments on different days. In each independent experiment, the assay was performed in triplicate wells, which were considered technical replicates. Thus, the experiments comprised three biological replicates, each with three technical replicates. The mean of the three technical replicates of each independent experiment was used as the experimental unit, so that n = 3 for all statistical comparisons.

## 5. Conclusions

Following a rigorous selection process, three strains were identified as *L. fermentum* VM016, VM023 and VM030, selected from the 142 initially isolated from cheese whey. These strains showed promising probiotic potential, exhibiting good co-aggregation with pathogenic bacteria, antioxidant and antifungal activity, tolerance to acid and bile salt challenges and promising technological aptitude, which suggests potential technological suitability for food applications, although this remains to be confirmed in an actual food matrix. The cytotoxicity and cytoprotection assays revealed promising in vitro results, as the strains protected both epithelial cell lines against the oxidative stressor, suggesting a potential beneficial effect that warrants further in vivo validation before any implications for consumer health can be established. Additional studies, including MIC-based antibiotic susceptibility testing and screening for resistance determinants, genome-based identification, food matrix incorporation and in vivo trials are therefore required to confirm these beneficial properties. Among the three candidates, *L. fermentum* VM016 exhibited the most favorable overall in vitro probiotic potential, although this qualitative ranking should be confirmed with an integrated scoring approach, as it did not outperform the other two strains in every assay.

## Figures and Tables

**Table 1:** Preliminary antibiotic susceptibility screening of the three selected isolates determined by disk diffusion (n = 3).

Antibiotic	Isolates		
	VM016	VM023	VM030
Vancomycin	Sensitive	Sensitive	Resistant
Ciprofloxacin	Sensitive	Sensitive	Sensitive
Amoxicillin/clavulanic acid	Sensitive	Sensitive	Sensitive
Penicillin	Sensitive	Sensitive	Sensitive
Oxacillin	Sensitive	Sensitive	Resistant
Clindamycin	Sensitive	Resistant	Sensitive
Rifampicin	Sensitive	Sensitive	Sensitive
Gentamicin	Resistant	Resistant	Resistant
Trimethoprim/sulfamethoxazole	Sensitive	Resistant	Resistant
Erythromycin	Sensitive	Sensitive	Sensitive

**Table 2:** Antimicrobial activity of the three *L. fermentum* strains against pathogenic bacteria.

Indicator Strain	*L. fermentum*		
	VM016	VM023	VM030
*E. coli* 81382	I	N	N
*E. coli* 81749	I	N	N
*E. coli* ATCC 35218	I	N	I
*Salmonella* sp.	I	I	N
*Salmonella* LM001	N	N	N
*Salmonella* LM006	I	I	N
*S. aureus* LM005	I	I	I
*S. aureus* ATCC 65389	I	I	I
*B. cereus* ATCC 11771	N	N	N

I: growth inhibition; N: no growth inhibition. Results are shown for n = 3.

**Table 3:** Co-aggregation ability (%) of the three *L. fermentum* strains with pathogenic bacteria.

Pathogenic Strains	*L. fermentum*		
	VM016	VM023	VM030
*E. coli* 81382	N	N	N
*E. coli* 81749	N	N	N
*E. coli* ATCC 35218	N	N	14.00 ± 4.10
*Salmonella* sp.	31.55 ± 3.12	N	N
*Salmonella* LM001	N	52.74 ± 3.00	7.69 ± 1.65
*Salmonella* LM006	N	N	N
*S. aureus* LM005	N	N	23.33 ± 5.50
*S. aureus* ATCC 65389	8.54 ± 4.20	10.17 ± 2.40	12.20 ± 3.00
*B. cereus* ATCC 11771	3.00 ± 1.26	N	N

N: no co-aggregation. Results are shown as mean ± SD (standard deviation) (n = 3).

**Table 4:** Antifungal activity of the three *L. fermentum* strains.

Fungal Stain	*L. fermentum*		
	VM016	VM023	VM030
*Aspergillus niger*	++	+	−
*Rhizopus* sp.	++	+	−
*Penicillium roqueforti*	−	−	−

(−) does not inhibit; (+) partially inhibits (diameter inhibition zone 0 to 10 mm); (++) totally inhibits (diameter inhibition zone > 10 mm). Results correspond to three independent experiments.

**Table 5:** Technological properties of the three selected *L. fermentum* strains expressed as growth index (GI) categories.

Assays	Condition	*L. fermentum*		
		VM016	VM023	VM030
Proteolytic activity	−	Absence	Absence	Absence
Lipolytic activity	−	Absence	Absence	Absence
Sugar tolerance	10% *w*/*v*	++	+	+
	15% *w*/*v*	++	+	+
	20% *w*/*v*	++	+	+
	25% *w*/*v*	++	+	+
NaCl tolerance	2% *w*/*v*	++	++	++
	4% *w*/*v*	++	++	++
CaCl_2_ tolerance	1% *w*/*v*	++	++	++
	2% *w*/*v*	++	++	++
	4% *w*/*v*	++	++	++
	6.5% *w*/*v*	++	++	++
pH tolerance	4	++	++	+
	5	++	++	+
	6	++	++	+
Growth temperature	8 °C	No	No	No
	20 °C	Yes	Yes	Yes
	50 °C	No	No	No
Essences tolerance	Vanilla 0.1% (*v*/*v*)	Yes	Yes	Yes
	Strawberry 0.1% (*v*/*v*)	Yes	Yes	Yes

(−) GI < 25%: strong inhibition; (+) 25% < GI < 75%: moderate growth; (++) GI > 75%: growth comparable to the control. Results correspond to three independent experiments (n = 3).

## Data Availability

The data which support the findings of this study are available from the corresponding author upon request.

## References

[1] Duarte P., Teixeira M., Silva S.C.E. (2021). Healthy Eating as a Trend: Consumers’ Perceptions towards Products with Nutrition and Health Claims. Rev. Bras. Gestão Negócios.

[2] Cardoso C.E.d.F., Silva S.T., Trindade M.E.F., Campos M.d.B.E., Cruz A.G., Lobo F.A.T.F., Teodoro A.J. (2025). Flavors of the Earth: Bioprospecting and Potential of Agricultural Ingredients in Yogurt Production with a Focus on Sustainability, Quality, and Technological Innovation. Foods.

[3] Bintsis T., Papademas P. (2023). Sustainable Approaches in Whey Cheese Production: A Review. Dairy.

[4] Ballatore M.B., Bettiol M.d.R.d.R., Vanden Braber N.L., Aminahuel C.A., Rossi Y.E., Petroselli G., Erra-Balsells R., Cavaglieri L.R., Montenegro M.A. (2020). Antioxidant and Cytoprotective Effect of Peptides Produced by Hydrolysis of Whey Protein Concentrate with Trypsin. Food Chem..

[5] Hill C., Guarner F., Reid G., Gibson G.R., Merenstein D.J., Pot B., Morelli L., Canani R.B., Flint H.J., Salminen S. (2014). The International Scientific Association for Probiotics and Prebiotics Consensus Statement on the Scope and Appropriate Use of the Term Probiotic. Nat. Rev. Gastroenterol. Hepatol..

[6] Paul C., Mishu I.D., Miah M.I., Bari M.L., Rahman S.R., Malek M.A. (2023). Isolation, Identification and Probiotic Potential of Lactic Acid Bacteria and Yeasts from Commercial Yogurt and Homemade Non-Dairy Fermented Food “KANJI.”. Int. J. Gastron. Food Sci..

[7] Soleimani H., Shokri R., Nami Y., Khandaghi J., Panahi B. (2023). Potential Probiotic Characterization of Lactic Acid Bacteria Isolated from Duimaj, an Iranian Traditional Snack Food, Using Biochemical, Molecular and Computational Approaches. LWT.

[8] Ale E.C., Ibáñez R.A., Wilbanks D.J., Peralta G.H., Ceylan F.D., Binetti A.G., Bolling B.W., Lucey J.A. (2023). Technological Role and Metabolic Profile of Two Probiotic EPS-Producing Strains with Potential Application in Yoghurt: Impact on Rheology and Release of Bioactive Peptides. Int. Dairy J..

[9] Ksiezarek M., Grosso F., Ribeiro T.G., Peixe L. (2022). Genomic Diversity of Genus Limosilactobacillus. Microb. Genom..

[10] D’ambrosio S., Ventrone M., Fusco A., Casillo A., Dabous A., Cammarota M., Corsaro M.M., Donnarumma G., Schiraldi C., Cimini D. (2022). *Limosilactobacillus fermentum* from Buffalo Milk Is Suitable for Potential Biotechnological Process Development and Inhibits Helicobacter Pylori in a Gastric Epithelial Cell Model. Biotechnol. Rep..

[11] Hao H., Nie Z., Wu Y., Liu Z., Luo F., Deng F., Zhao L. (2024). Probiotic Characteristics and Anti-Inflammatory Effects of *Limosilactobacillus fermentum* 664 Isolated from Chinese Fermented Pickles. Antioxidants.

[12] Kanagaraj N., Duraisamy S., Balakrishnan S., Narayanapillai U., Ramasamy G. (2012). Characterization of Bacteriocin Producing Lactic Acid Bacteria and Its Application as a Food Preservative. Afr. J. Microbiol. Res..

[13] Marasco R., Gazzillo M., Campolattano N., Sacco M., Muscariello L. (2022). Isolation and Identification of Lactic Acid Bacteria from Natural Whey Cultures of Buffalo and Cow Milk. Foods.

[14] De Chiara I., Marasco R., Della Gala M., Fusco A., Donnarumma G., Muscariello L. (2024). Probiotic Properties of *Lactococcus lactis* Strains Isolated from Natural Whey Starter Cultures. Foods.

[15] Melia S., Juliyarsi I., Kurnia Y.F., Rossi E., Alzahra H. (2022). Selection of Potential Lactic Acid Bacteria as a Candidate Probiotic on Cheese Making by Product Whey. Adv. Anim. Vet. Sci..

[16] De Chiara I., Marasco R., Della Gala M., Alfano A., Parecha D., Costanzo N., Schiraldi C., Muscariello L. (2025). Isolation and Partial Characterization of Lactic Acid Bacteria from Natural Whey Starter Culture. Fermentation.

[17] Tuo Y., Yu H., Ai L., Wu Z., Guo B., Chen W. (2013). Aggregation and Adhesion Properties of 22 Lactobacillus Strains. J. Dairy Sci..

[18] Aziz G., Fakhar H., Rahman S.U., Tariq M., Zaidi A. (2019). An Assessment of the Aggregation and Probiotic Characteristics of Lactobacillus Species Isolated from Native (Desi) Chicken Gut. J. Appl. Poult. Res..

[19] Rodríguez-Sánchez S., Fernández-Pacheco P., Seseña S., Pintado C., Palop M.L. (2021). Selection of Probiotic Lactobacillus Strains with Antimicrobial Activity to Be Used as Biocontrol Agents in Food Industry. LWT.

[20] Nami Y., Vaseghi Bakhshayesh R., Manafi M., Hejazi M.A. (2019). Hypocholesterolaemic Activity of a Novel Autochthonous Potential Probiotic *Lactobacillus plantarum* YS5 Isolated from Yogurt. LWT.

[21] Zhou H., Wang S., Liu W., Chang L., Zhu X., Mu G., Qian F. (2023). Probiotic Properties of *Lactobacillus paraplantarum* LS-5 and Its Effect on Antioxidant Activity of Fermented Sauerkraut. Food Biosci..

[22] Marquez A., Andrada E., Russo M., Bolondi M.L., Fabersani E., Medina R., Gauffin-Cano P. (2022). Characterization of Autochthonous Lactobacilli from Goat Dairy Products with Probiotic Potential for Metabolic Diseases. Heliyon.

[23] Terpou A., Papadaki A., Lappa I.K., Kachrimanidou V., Bosnea L.A., Kopsahelis N. (2019). Probiotics in Food Systems: Significance and Emerging Strategies Towards Improved Viability and Delivery of Enhanced Beneficial Value. Nutrients.

[24] Okaiyeto S.A., Sutar P.P., Chen C., Ni J.B., Wang J., Mujumdar A.S., Zhang J.S., Xu M.Q., Fang X.M., Zhang C. (2024). Antibiotic Resistant Bacteria in Food Systems: Current Status, Resistance Mechanisms, and Mitigation Strategies. Agric. Commun..

[25] Duche R.T., Singh A., Wandhare A.G., Sangwan V., Sihag M.K., Nwagu T.N.T., Panwar H., Ezeogu L.I. (2023). Antibiotic Resistance in Potential Probiotic Lactic Acid Bacteria of Fermented Foods and Human Origin from Nigeria. BMC Microbiol..

[26] Islam S., Biswas S., Jabin T., Moniruzzaman M., Biswas J., Uddin M.S., Akhtar-E-Ekram M., Elgorban A.M., Ghodake G., Syed A. (2023). Probiotic Potential of *Lactobacillus plantarum* DMR14 for Preserving and Extending Shelf Life of Fruits and Fruit Juice. Heliyon.

[27] Vieira de Souza F., Roque R., Silva Moreira J.L., Resende de Souza M., Nicoli J.R., Neumann E., Cantini Nunes Á. (2012). Transfer of Antibiotic Resistance Determinants between Lactobacilli Isolates from the Gastrointestinal Tract of Chicken. Benef. Microbes.

[28] Rzepkowska A., Zielińska D., Ołdak A., Kołożyn-Krajewska D. (2017). Organic Whey as a Source of Lactobacillus Strains with Selected Technological and Antimicrobial Properties. Int. J. Food Sci. Technol..

[29] Xu Y.B., Li D.L., Ding X.Q., Wang Y.Y., Liang S., Xie L.Y., Zhang Y.F., Fu A.K., Yu W.Q., Zhan X.A. (2023). Probiotic Characterization and Comparison of Broiler-Derived Lactobacillus Strains Based on Technique for Order Preference by Similarity to Ideal Solution Analysis. Poult. Sci..

[30] Mkadem W., Belguith K., Oussaief O., ElHatmi H., Indio V., Savini F., De Cesare A., Boudhrioua N. (2023). Systematic Approach to Select Lactic Acid Bacteria from Spontaneously Fermented Milk Able to Fight Listeria Monocytogenes and Staphylococcus Aureus. Food Biosci..

[31] Liang N., Zhao Z., Curtis J.M., Gänzle M.G. (2022). Antifungal Cultures and Metabolites of Lactic Acid Bacteria for Use in Dairy Fermentations. Int. J. Food Microbiol..

[32] Ayyash M., Abu-Jdayil B., Itsaranuwat P., Almazrouei N., Galiwango E., Esposito G., Hunashal Y., Hamed F., Najjar Z. (2020). Exopolysaccharide Produced by the Potential Probiotic *Lactococcus garvieae* C47: Structural Characteristics, Rheological Properties, Bioactivities and Impact on Fermented Camel Milk. Food Chem..

[33] Reissig Soares Vitola H., da Silva Dannenberg G., de Lima Marques J., Völz Lopes G., Padilha da Silva W., Fiorentini Â.M. (2018). Probiotic Potential of *Lactobacillus casei* CSL3 Isolated from Bovine Colostrum Silage and Its Viability Capacity Immobilized in Soybean. Process Biochem..

[34] Kim S., Lee J.Y., Jeong Y., Kang C.H. (2022). Antioxidant Activity and Probiotic Properties of Lactic Acid Bacteria. Fermentation.

[35] Liu R., Li S., Yang B., Chen L., Ge Q., Xiong G., Yu H., Wu M., Zhang W. (2021). Investigation of the Antioxidant Capacity of Cell-Free Extracts from *Lactobacillus plantarum* NJAU-01 Obtained by Different Cell Disruption Methods. LWT.

[36] Vitheejongjaroen P., Kanthawang P., Loison F., Jaisin Y., Pachekrepapol U., Taweechotipatr M. (2021). Antioxidant Activity of Bifidobacterium Animalis MSMC83 and Its Application in Set-Style Probiotic Yoghurt. Food Biosci..

[37] Aktepe Y., Aydın F., Bozoğlu T., Özer G., Çakır İ. (2024). Molecular Characterization and Multifunctional Evaluation of Lactic Acid Bacteria Isolated from Traditional Sourdough: Multifunctional Lactic Acid Bacteria. Int. J. Food Microbiol..

[38] Braïek O.B., Morandi S., Cremonesi P., Smaoui S., Hani K., Ghrairi T. (2018). Safety, Potential Biotechnological and Probiotic Properties of Bacteriocinogenic *Enterococcus lactis* Strains Isolated from Raw Shrimps. Microb. Pathog..

[39] Abdillahi H.S., Verschaeve L., Finnie J.F., Van Staden J. (2012). Mutagenicity, Antimutagenicity and Cytotoxicity Evaluation of South African Podocarpus Species. J. Ethnopharmacol..

[40] Aherne S.A., O’brien N.M. (2000). Mechanism of Protection by the Flavonoids, Quercetin and Rutin, against Tert-Butylhydroperoxide. and Menadione- Induced DNA Single Strand Breaks in Caco-2 Cells. Free Radic. Biol. Med..

[41] Rossi Y.E., Bohl L.P., Vanden Braber N.L., Ballatore M.B., Escobar F.M., Bodoira R., Maestri D.M., Porporatto C., Cavaglieri L.R., Montenegro M.A. (2020). Polyphenols of Peanut (*Arachis hypogaea* L.) Skin as Bioprotectors of Normal Cells. Studies of Cytotoxicity, Cytoprotection and Interaction with ROS. J. Funct. Foods.

[42] Naser S.M., Dawyndt P., Hoste B., Gevers D., Vandemeulebroecke K., Cleenwerck I., Vancanneyt M., Swings J. (2007). Identification of Lactobacilli by PheS and RpoA Gene Sequence Analyses. Int. J. Syst. Evol. Microbiol..

[43] (2012). EFSA Panel on Additives and Products or Substances used in Animal Feed (FEEDAP). Guidance on the Assessment of Bacterial Susceptibility to Antimicrobials of Human and Veterinary Importance. EFSA J..

[44] Abushelaibi A., Al-Mahadin S., El-Tarabily K., Shah N.P., Ayyash M. (2017). Characterization of Potential Probiotic Lactic Acid Bacteria Isolated from Camel Milk. LWT-Food Sci. Technol..

[45] Mohd-Zubri N.S., Ramasamy K., Abdul-Rahman N.Z. (2022). Characterization and Potential Oral Probiotic Properties of *Lactobacillus plantarum* FT 12 and Lactobacillus Brevis FT 6 Isolated from Malaysian Fermented Food. Arch. Oral Biol..

[46] Díaz-Vergara L., Pereyra C.M., Montenegro M., Pena G.A., Aminahuel C.A., Cavaglieri L.R. (2017). Encapsulated Whey–Native Yeast *Kluyveromyces marxianus* as a Feed Additive for Animal Production. Food Addit. Contam. Part A Chem. Anal. Control Expo. Risk Assess..

[47] Prabhurajeshwar C., Chandrakanth K. (2019). Evaluation of Antimicrobial Properties and Their Substances against Pathogenic Bacteria In-Vitro by Probiotic Lactobacilli Strains Isolated from Commercial Yoghurt. Clin. Nutr. Exp..

[48] Loyeau P.A., Spotti M.J., Vanden Braber N.L., Rossi Y.E., Montenegro M.A., Vinderola G., Carrara C.R. (2018). Microencapsulation of Bifidobacterium Animalis Subsp. Lactis INL1 Using Whey Proteins and Dextrans Conjugates as Wall Materials. Food Hydrocoll..

[49] Zhu Y., Xu Y., Yang Q. (2021). Antifungal Properties and AFB1 Detoxification Activity of a New Strain of *Lactobacillus plantarum*. J. Hazard. Mater..

[50] Abid Y., Casillo A., Gharsallah H., Joulak I., Lanzetta R., Corsaro M.M., Attia H., Azabou S. (2018). Production and Structural Characterization of Exopolysaccharides from Newly Isolated Probiotic Lactic Acid Bacteria. Int. J. Biol. Macromol..

[51] Quaroni A., Isselbacher K.J. (1981). Cytotoxic Effects and Metabolism of Benzo[a]pyrene and 7,12-Dimethylbenz[a]anthracene in Duodenal and Ileal Epithelial Cell Cultures. J. Natl. Cancer Inst..

[52] Turco L., Catone T., Caloni F., Di Consiglio E., Testai E., Stammati A. (2011). Caco-2/TC7 Cell Line Characterization for Intestinal Absorption: How Reliable Is This in Vitro Model for the Prediction of the Oral Dose Fraction Absorbed in Human?. Toxicol. Vitr..

[53] Repetto G., del Peso A., Zurita J.L. (2008). Neutral Red Uptake Assay for the Estimation of Cell Viability/ Cytotoxicity. Nat. Protoc..

[54] Zarour K., Prieto A., Pérez-Ramos A., Kihal M., López P. (2018). Analysis of Technological and Probiotic Properties of Algerian *L. mesenteroides* Strains Isolated from Dairy and Non-Dairy Products. J. Funct. Foods.

[55] Ishaq M., Ali N., Ullah I., Ullah S., Farhan M., Bashar K.U., Rahman H. (2019). Biochemical Identification of Lactobacilli from Chicken Intestine and Their Potential Probiotic Activity. Pesqui. Agropecu. Bras..

